# Inert coupling of IRDye800CW to monoclonal antibodies for clinical optical imaging of tumor targets

**DOI:** 10.1186/2191-219X-1-31

**Published:** 2011-12-01

**Authors:** Ruth Cohen, Marieke A Stammes, Inge HC de Roos, Marijke Stigter-van Walsum, Gerard WM Visser, Guus AMS van Dongen

**Affiliations:** 1 Department of Otolaryngology/Head and Neck Surgery, VU University Medical Center, De Boelelaan 1117, P.O. Box 7057, Amsterdam, 1007 MB, The Netherlands; 2 Department of Nuclear Medicine & PET Research, VU University Medical Center, De Boelelaan 1117, P.O. Box 7057, Amsterdam, 1007 MB, The Netherlands

**Keywords:** zirconium-89, monoclonal antibodies, IRDye800CW, cetuximab, bevacizumab

## Abstract

**Background:**

Photoimmunodetection, in which monoclonal antibodies [mAbs] are labeled with fluorescent dyes, might have clinical potential for early detection and characterization of cancer. For this purpose, the dye should be coupled in an inert way to mAb. In this study, different equivalents of IRDye800CW, a near-infrared fluorescent dye, were coupled to ^89^Zr-labeled cetuximab and bevacizumab, and conjugates were evaluated in biodistribution studies. Radiolabeled mAbs were used to allow accurate quantification for assessment of the number of dye groups that can be coupled to mAbs without affecting their biological properties.

**Methods:**

^89^Zr-cetuximab and ^89^Zr-bevacizumab, containing 0.5 ^89^Zr-desferal group per mAb molecule, were incubated with 1 to 10 eq IRDye800CW at pH 8.5 for 2 h at 35°C, and ^89^Zr-mAb-IRDye800CW conjugates were purified by a PD10 column using 0.9% NaCl as eluent. HPLC analysis at 780 nm was used to assess conjugation efficiency. *In vitro *stability measurements were performed in storage buffer (0.9% NaCl or PBS) at 4°C and 37°C and human serum at 37°C. ^89^Zr-mAb-IRDye800CW conjugates and ^89^Zr-mAb conjugates (as reference) were administered to nude mice bearing A431 (cetuximab) or FaDu (bevacizumab) xenografts, and biodistribution was assessed at 24 to 72 h after injection.

**Results:**

Conjugation efficiency of IRDye800CW to ^89^Zr-mAbs was approximately 50%; on an average, 0.5 to 5 eq IRDye800CW was conjugated. All conjugates showed optimal immunoreactivity and were > 95% stable in storage buffer at 4°C and 37°C and human serum at 37°C for at least 96 h. In biodistribution studies with ^89^Zr-cetuximab-IRDye800CW, enhanced blood clearance with concomitant decreased tumor uptake and increased liver uptake was observed at 24 to 72 h post-injection when 2 or more eq of dye had been coupled to mAb. No significant alteration of biodistribution was observed 24 to 48 h after injection when 1 eq of dye had been coupled. ^89^Zr-bevacizumab-IRDye800CW showed a similar tendency, with an impaired biodistribution when 2 eq of dye had been coupled to mAb.

**Conclusion:**

Usage of ^89^Zr-mAbs allows accurate quantification of the biodistribution of mAbs labeled with different equivalents of IRDye800CW. Alteration of biodistribution was observed when more than 1 eq of IRDye800CW was coupled to mAbs.

## Background

Molecular imaging with monoclonal antibodies [mAbs] harbors a potential for diagnosis and therapy response evaluation, as well as for the evaluation of molecular processes *in vivo*. In addition, it can be used to speed up and guide mAb development and to tailor therapy with existing mAbs by providing information about the targeting performance of mAbs and the expression status of cell surface targets. The mAbs labeled with radionuclides can be used for single photon emission computed tomography [SPECT] or positron-emission tomography [PET] and are particularly well suited for a whole-body quantitative imaging of deep-seated tissues. To this end, we recently introduced clinical immuno-PET, which is like performing a 'comprehensive immunohistochemical staining *in vivo*' [1,2]. Procedures were developed to radiolabel intact mAbs in a clinical good manufacturing practice [cGMP]-compliant way with zirconium-89 (^89^Zr, *t*_1/2 _= 78.4 h) and iodine-124 (^124^I, *t*_1/2 _= 100.3 h), enabling a broadscale clinical application of immuno-PET [3-6]. Notwithstanding these promising developments, immuno-PET has a limited resolution.

Photoimmunodetection [PID], in which mAbs are labeled with fluorescent dyes, might have a complementary clinical potential to immuno-PET [7-19]. It allows high-resolution, real-time, dynamic imaging of superficial tissue layers at the cellular level, without radiation burden to the patient. Therefore, it might be ideal for the detection and characterization of an early-stage or residual disease, for example of cancer during surgery or in a screening setting. During the past years, the preclinical exploration of PID has been boosted by the introduction of more advanced fluorescent dyes, which emit in the near-infrared [NIR] (approximately 700 to 1,000 nm) region of the spectrum [20]. The advantage of NIR dyes is that they enable reasonable tissue penetration of exciting and emitted lights, while the amount of autofluorescence is negligible [21]. Nevertheless, PID is still waiting for a broadscale clinical application.

The only Food and Drug Administration [FDA]-approved NIR fluorophore until now is indocyanine green [ICG]. It was approved by the FDA in 1958. However, since the ICG molecule itself cannot be covalently coupled to mAbs, a modified version containing an *N*-hydroxysuccinimide [NHS] ester-designated ICG-sulfo-OSu was developed in 1995 by Ito et al. [22]. Although conjugation of this ICG dye to proteins appeared facile, a serious loss of fluorescence was observed upon binding to a protein [22]; albeit with internalizing mAbs, it might still be applicable [23]. A promising next-generation NIR fluorophore is IRDye^®^800CW [24]. This NIR dye can be functionalized with either an NHS or a maleimide reactive group, allowing its attachment to a broad spectrum of targeting biomolecules. This dye has been evaluated in several preclinical studies [25-28], but before being used in clinical investigations, it must undergo rigorous toxicity testing, the first stage of which must be conducted in animals. Such studies in male and female rats revealed no pathological evidence of toxicity after a single intravenous administration of IRDye800CW at dose levels of 1, 5, and 20 mg/kg or 20 mg/kg intradermally [29].^a^

A prerequisite for using tracer-labeled mAbs in clinical immuno-PET or PID is that the radionuclide or dye is coupled to the mAb in an inert way, which means that the binding characteristics, pharmacokinetics, and dynamics of the mAb do not become impaired upon coupling of these tracers. While the stability, binding characteristics, and *in vivo *biodistribution of radioimmunoconjugates can easily and accurately be analyzed in a quantitative way, this is much more challenging for photoimmunoconjugates. This made us decide to use ^89^Zr-labeled mAbs as a starting point to facilitate analytical procedures to study, analyze, and quantify the inertness of dye coupling to mAbs. At a later stage, such conjugates might be applied in a multimodal imaging approach, in which PET is used for a low-resolution whole-body analysis and PID for an additional local, high-resolution diagnostic evaluation.

For these studies, we selected the US FDA-approved mAbs cetuximab (Erbitux; Merck, Darmstadt, Germany) and bevacizumab (Avastin; Genentech, Inc., South San Francisco, CA, USA/Hoffmann-La Roche Inc., Penzberg, Germany), directed against the epidermal growth factor receptor [EGFR] and the vasculature epidermal growth factor [VEGF], respectively, as the model mAbs. Altered expressions of EGFR and VEGF are early steps in the development of many cancers; therefore, these are appealing targets for early tumor detection by PID. Both cetuximab and bevacizumab have been tested as radioimmunoconjugates in combination with ^89^Zr in preclinical [30,31] as well as ongoing clinical immuno-PET studies.

In this study, ^89^Zr-labeled cetuximab and bevacizumab are modified with, on an average, 0.5 to 5 eq of IRDye800CW per mAb molecule. The integrity and immunoreactivity of these conjugates are also assessed after storage at 4°C and 37°C in a buffer or in human serum at 37°C. In addition, comparative biodistribution and optical imaging studies are presented.

## Methods

### Materials

The mAb cetuximab (Erbitux; 5 mg/mL) was purchased from Merck, and bevacizumab (Avastin; 25 mg/mL), from Hoffmann-La Roche Inc. The human squamous cell carcinoma cell line A431 was obtained from the American Type Culture Collection (ATCC number CRL-15555), and the head and neck squamous cell cancer line FaDu, from Karl-Heinz Heider (Boehringer Ingelheim, Vienna, Austria). IRDye800CW-NHS ester (MW 1,166 Da, LI-COR Biosciences) was supplied by Westburg BV, Leusden, The Netherlands. ^89^Zr (*t*_1/2 _= 78.4 h) was purchased from IBA Molecular (Louvain-la-Neuve, Belgium) as [^89^Zr]Zr-oxalate in 1.0 M oxalic acid (≥ 0.15 GBq/nmol) [32].

### Radiolabeling of cetuximab or bevacizumab

Antibody premodification and subsequent labeling with ^89^Zr were performed as described previously, using desferal [Df] (desferrioxamine B, Novartis Pharma BV, Arnhem, The Netherlands) as the chelate [33] (see Additional file 1). When cetuximab was used, it was buffer-exchanged on a PD10 column (GE Healthcare Life Sciences, Eindhoven, The Netherlands) to a solution of 0.9% NaCl before chelate conjugation. Bevacizumab was used directly from the vial.

### Conjugation of IRDye800CW to ^89^Zr-mAbs

**Figure 1:**
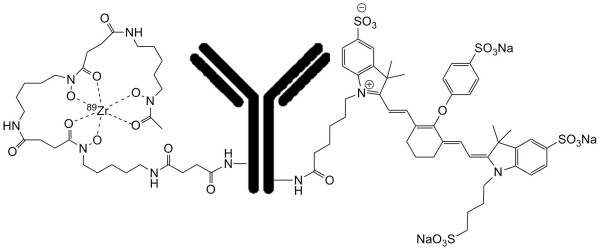
**Schematic representation of ^89^Zr-mAb-IRDye800CW**.

For the conjugation of IRDye800CW to ^89^Zr-cetuximab/bevacizumab, the solution was brought to pH 8.5 by adding 0.1 M Na_2_CO_3_. Subsequently, 20 μL of IRDye800CW, diluted in dimethyl sulfoxide, was added, and the total volume was adjusted to 1 mL with 0.9% NaCl. The IRDye800CW was added to the mAb solution at a 10:1 to 1:1 molar ratio. The reaction mixture was incubated for 2 h at 35°C in a thermomixer at 550 rpm. The unreacted dye was removed by purification of the conjugates on a PD10 column, using 0.9% NaCl as eluent. The flow through and the first 1.5 mL were discarded. The next 2 mL containing the conjugated mAb was collected. For a schematic representation of ^89^Zr-mAb-IRDye800CW, see Figure 1.

### Analyses

Conjugates were analyzed by instant thin-layer chromatography [ITLC] for radiochemical purity, by high-performance liquid chromatography [HPLC] for mAb integrity and purity, and by an antigen-binding assay for immunoreactivity. ITLC analysis was performed on silica gel-impregnated glass fiber sheets (PI Medical Diagnostic Equipment BV, Tijnje, The Netherlands), with a 20-mM citrate buffer of pH 5.0 as the mobile phase. HPLC analysis was performed on a JASCO Benelux BV HPLC (de Meern, The Netherlands) with a diode array detector system and an inline radiodetector (Raytest Isotopenmessgeräte GmbH, Straubenhardt, Germany) using a Superdex 200 10/300 GL size exclusion column (GE Healthcare Life Sciences). The eluent consisted of 0.05 M sodium phosphate/0.15 M sodium chloride plus 0.05% sodium azide (pH 6.8), and the flow was set at a rate of 0.5 mL/min. HPLC measurements were performed at *A *= 280 nm to measure mAb absorption, at *A *= 430 nm to measure the absorption of *N*-sucDf-Fe(III), and at *A *= 780 nm to measure the absorption of IRDye800CW. The chelate-to-mAb and IRDye800CW-to-mAb molar ratios were determined by HPLC, using the areas under the curve at *A*_280_, *A*_430_, and/or *A*_780_.

*In vitro *binding characteristics were determined in an immunoreactivity assay essentially as described before [30], using A431 cells fixed with 2% paraformaldehyde for cetuximab conjugates. For bevacizumab, an enzyme-linked immunosorbent assay [ELISA] was used, adapted from Nagengast et al. [31]. Binding data were graphically analyzed in a modified Lineweaver-Burk (double-reciprocal) plot, and the immunoreactive fraction was determined by linear extrapolation to conditions representing infinite antigen excess.

### Serum stability test

Serum stability tests were performed with cetuximab/bevacizumab-IRDye800CW conjugates, with different equivalents of dye coupled to cetuximab/bevacizumab. Cetuximab/bevacizumab-IRDye800CW and human serum at a ratio of 1:1 (*v/v*) and 1% sodium azide were filter-sterilized, mixed, and incubated in a 12-well plate at 37°C and 5% CO_2_. Control samples were incubated in sterile phosphate-buffered saline [PBS] instead of human serum. For analysis of stability, samples were diluted threefold in PBS prior to HPLC analysis.

### Biodistribution

For evaluation of the biodistribution of ^89^Zr-cetuximab/bevacizumab-IRDye800CW and ^89^Zr-cetuximab/bevacizumab conjugates, non-tumor-bearing female nude mice (Hsd athymic *nu/nu*, 25 to 32 g; Harlan Laboraties BV CPB, Boxmeer, The Netherlands) as well as mice bearing subcutaneously implanted A431 or FaDu tumors were used. All animal experiments were performed according to the Dutch National Institutes of Health principles of laboratory animal care and Dutch national law ('Wet op de dierproeven', Stb 1985, 336).

In a pilot biodistribution study, a total of 16 non-tumor-bearing mice were injected with 0.31 MBq of ^89^Zr-cetuximab-IRDye800CW or ^89^Zr-cetuximab or containing, on an average, 1.5, 2.5, or 5.0 eq of dye per mAb molecule (^89^Zr-cetuximab-IRDye800CW; 1.5, 2.5. or 5.0 eq). The mice received 100 μg cetuximab in a total volume of 150 μL intravenously. At 24 h after injection, the mice were anesthetized, bled, euthanized, and dissected.

In the next biodistribution study with cetuximab, a total of 60 A431-bearing mice with a tumor size of 168 ± 75 mm^3 ^were injected with 0.37 MBq of ^89^Zr-cetuximab-IRDye800CW (0.5, 1.0, or 2.0 eq) or ^89^Zr-cetuximab. The mice received 100 μg cetuximab in a total volume of 150 μL intravenously. At 24, 48, and 72 h after injection, five mice per group and time point were anesthetized, bled, euthanized, and dissected. For the biodistribution study with bevacizumab, a total of 30 FaDu-bearing mice with a tumor size of 600 ± 200 mm^3 ^were injected with 0.37 MBq of ^89^Zr-bevacizumab-IRDye800CW (1.0 or 2.0 eq) or ^89^Zr-bevacizumab. The mice received 40 μg bevacizumab in a total volume of 175 μL intravenously. At 24 h after injection, blood was collected from the tail vein of all mice. At 48 and 72 h, five mice per group and time point were anesthetized, bled, euthanized, and dissected. The blood, tumor, and organs were weighed, and the amount of radioactivity was measured in a γ-well counter (Wallac LKB-CompuGamma 1282; Pharmacia, Uppsala, Sweden). Radioactivity uptake was measured as the percentage of the injected dose per gram of tissue [%ID/g]. Differences in tissue uptake between conjugates were statistically analyzed for each time point with SPSS 15.0 (SPSS Inc., Chicago, IL, USA) using the Student's *t *test for independent samples. Two-sided significance levels were calculated, and *P *< 0.05 was considered statistically significant.

### *In vivo *fluorescence imaging

NIR images were acquired with the IVIS Lumina system with indocyanine green filter sets (Caliper Life Science, Hopkinton, MA, USA), as described before [34]. Data were analyzed with the Living Image software from xenogeny version 3.2 (Caliper Life Science). Imaging time was 1 s.

## Results

### Production and quality controls of ^89^Zr-cetuximab-IRDye800CW and ^89^Zr-bevacizumab-IRDye800CW

**Figure 2:**
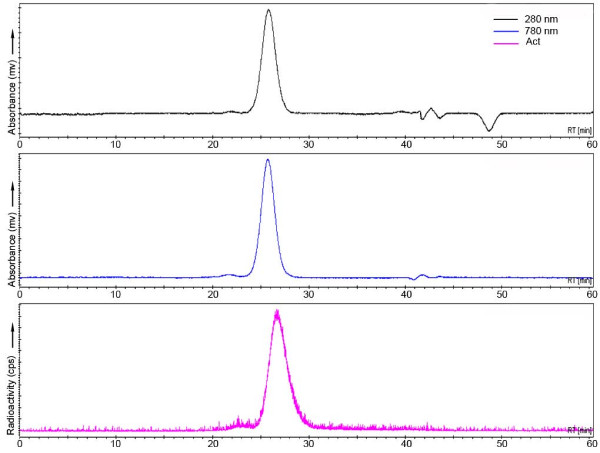
**HPLC chromatogram of ^89^Zr-cetuximab-IRDye800CW (3.5 eq)**. The upper two channels show the UV absorption of cetuximab at 280 nm and IRDye800CW at 780 nm at a retention time of 26 min. The lower channel represents the radioactive signal of the coupled ^89^Zr.

On an average, 0.5 group of Df was coupled to cetuximab or bevacizumab, while labeling with ^89^Zr resulted in an overall labeling yield of 75%. ITLC and HPLC showed that the radiochemical purity of the product always exceeded 95% after purification on PD10. Subsequent coupling of IRDye800CW to the radioactive conjugate gave conjugation efficiencies of about 50%, resulting in IRDye800CW-to-mAb molar ratios of 0.5:1 to 5:1, as assessed by HPLC analysis. After purification on PD10, the dual-labeled conjugate was found to be more than 99% pure for ^89^Zr as well as for IRDye800CW (Figure 2). The immunoreactivity of ^89^Zr-cetuximab was 99% at infinite antigen access and did not alter when up to 5 eq of IRDye800CW was coupled. The ELISA binding assay for ^89^Zr-bevacizumab gave a binding of 75%, which is optimal for this assay, and did not alter upon coupling of 1 of 2 eq of dye. HPLC analysis confirmed that there was no difference in the conjugation efficiency of IRDye800CW to cetuximab/bevacizumab or ^89^Zr-cetuximab/bevacizumab. ^89^Zr-cetuximab/bevacizumab-IRDye800CW conjugates could be stored in 0.9% NaCl at 4°C for at least 4 days (cetuximab) or at least 2 days (bevacizumab), without any loss of integrity and immunoreactivity as assessed by HPLC or binding assay.

**Figure 3:**
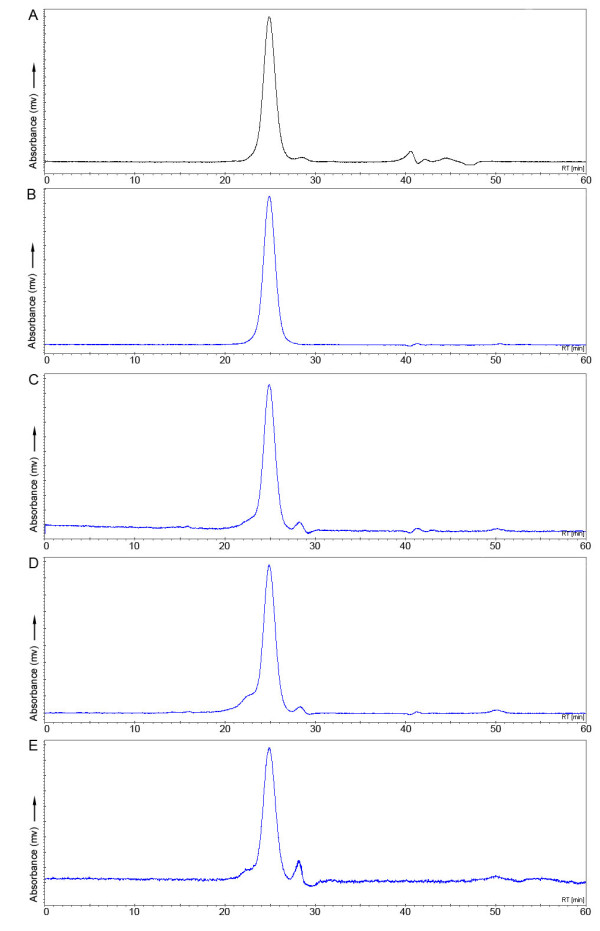
**HPLC chromatograms of serum incubations of cetuximab-IRDye800CW**. HPLC chromatograms at 280 nm (black line, **A**) and at 780 nm (blue lines, **B**-**E**) of cetuximab-IRDye800CW conjugates. Cetuximab-IRDye800CW (2.8 eq) incubated in PBS at 280 (A) and 780 nm (B). Cetuximab-IRDye800CW coupled with 1.5 (C), 2.8 (D), or 4.8 (E) eq of dye, incubated in serum for 96 h at 37°C days prior to HPLC analysis.

Cetuximab and bevacizumab conjugated with, on an average, 1 to 5 eq of dye were incubated in the presence of human serum at 37°C and in PBS at 37°C as reference, and HPLC profiles of the mAb at *A*_780 _were made to provide information on the physicochemical properties of the conjugate. None of the conjugates showed any instability upon storage in PBS for at least 96 h, as illustrated for cetuximab-IRDye (2.8 eq) in Figure 3A, B. In human serum, a minimal percentage of IRDye800CW was released from the antibody: 1.4% to 1.8% for cetuximab-IRDye (1.5, 2.8, and 4.8 eq) and 2.8% and 3.5% for bevacizumab-IRDye (1.1 and 2.2 eq). Besides this, only minor peak changes were observed for both mAbs, as illustrated for cetuximab-IRDye (Figure 3C, D, E).

### Biodistribution

**Figure 4:**
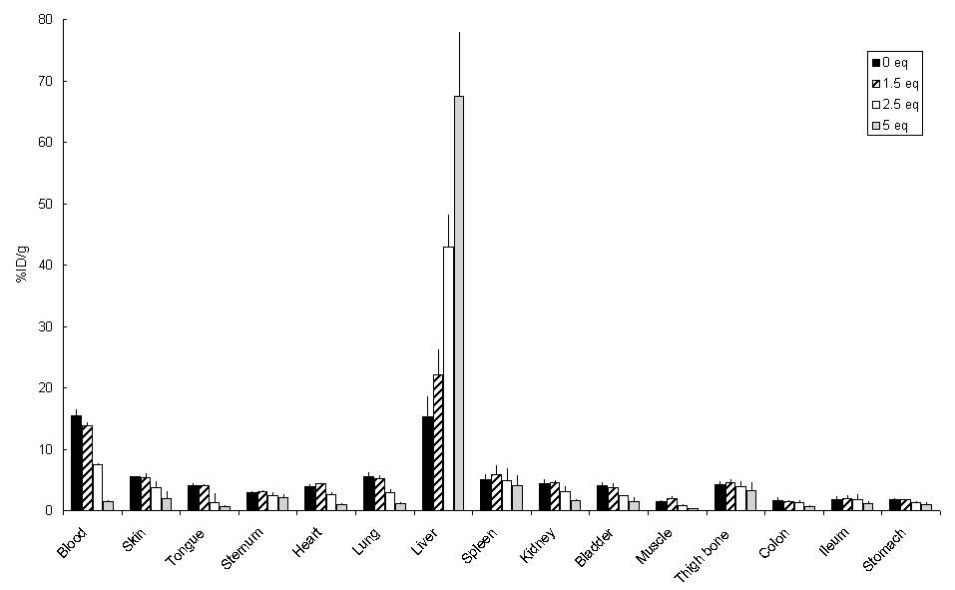
**Biodistribution of ^89^Zr-cetuximab-IRDye800CW in non-tumor-bearing mice**. Biodistribution of intravenously injected ^89^Zr-cetuximab and ^89^Zr-cetuximab-IRDye800CW (1.5, 2.5, and 5 eq) in non-tumor-bearing nude mice at 24 h after injection. Data are presented as %ID/g ± SD.

To get insight in the relationship between the number of dyes coupled to the mAb and its pharmacokinetics, a pilot biodistribution study was performed in non-tumor-bearing mice. Figure 4 shows the uptake in the blood and organs of mice injected with ^89^Zr-cetuximab or with ^89^Zr-cetuximab-IRDye800CW (1.5, 2.5, or 5.0 eq) at 24 h after injection. Blood levels were 15.4 ± 1.3, 13.8 ± 0.8, 7.4 ± 0.4, and 1.5 ± 0.3%ID/g for 0, 1.5, 2.5, and 5.0 eq of coupled dye, respectively. Liver uptake increased with increasing equivalents of dye: 15.3 ± 3.3, 22.2 ± 4.2, 42.9 ± 5.4, and 67.5 ± 10.5%ID/g, respectively. These data indicate that conjugates with 1.5 groups of dye show a tendency of altered biodistribution, while for conjugates with 2.5 or more groups of dye, the alteration is evident.

**Figure 5:**
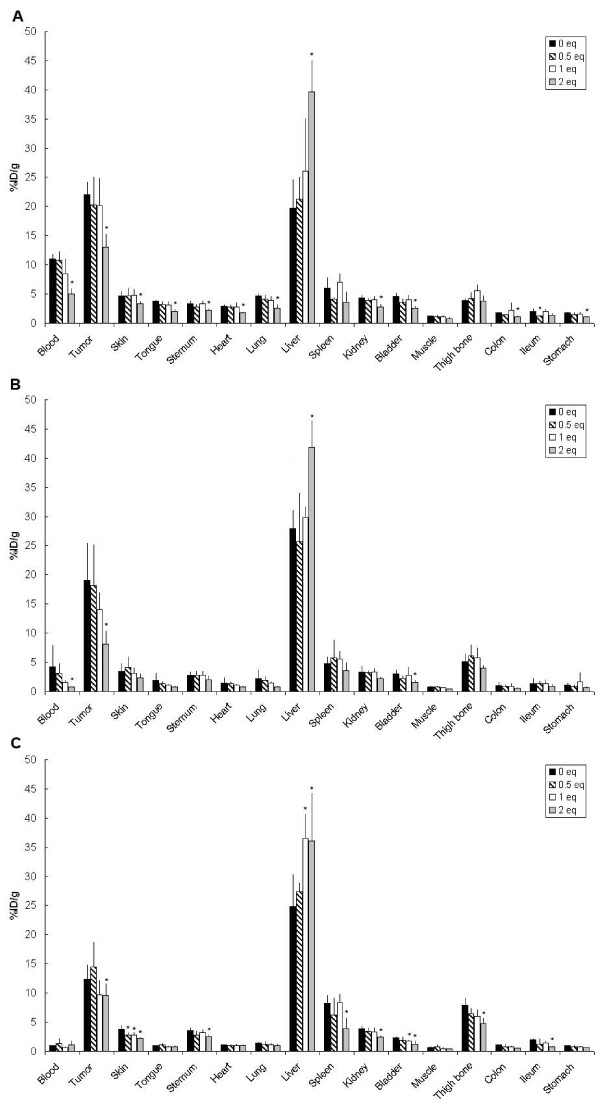
**Biodistribution of ^89^Zr-cetuximab-IRDye800CW in tumor-bearing mice**. Biodistribution of intravenously injected ^89^Zr-cetuximab and ^89^Zr-cetuximab-IRDye800CW (0.5, 1, and 2 eq) in A431 xenograft-bearing nude mice at 24 (**A**), 48 (**B**), and 72 (**C**) h after injection. Bars marked with an asterisk have an uptake that is significantly (*P *≤ 0.05) different from the uptake of ^89^Zr-cetuximab. Data are presented as %ID/g ± SD.

^89^Zr-cetuximab-IRDye800CW (0, 0.5, 1.0, or 2.0 eq) was administered to nude mice bearing A431 tumors to study the impact of IRDye800CW-to-mAb molar ratio on the biodistribution, tumor targeting included, in more detail (Figure 5). Blood levels of ^89^Zr-cetuximab coupled with 0, 0.5, 1.0, and 2.0 eq of dye at 24 h after injection were 11.0 ± 1.0, 10.8 ± 1.6, 8.5 ± 2.6, and 5.0 ± 1.0%ID/g, respectively (Figure 5A). The blood clearance of ^89^Zr-cetuximab-IRDye (2.0 eq) was significantly different from that of ^89^Zr-cetuximab-IRDye (0 eq). More rapid blood clearance upon more coupled groups of dye was accompanied by increasing liver uptake (19.8 ± 5.0, 21.3 ± 3.9, 26.1 ± 9.1, and 39.6 ± 5.4%ID/g for 0, 0.5, 1.0, and 2.0 eq coupled, respectively) and decreasing tumor uptake (22.0 ± 2.2, 20.2 ± 5.0, 20.2 ± 4.8, and 13.0 ± 2.4%ID/g, respectively). ^89^Zr-cetuximab-IRDye800CW (2.0 eq) also showed decreased uptake in some of the normal organs, among which are the skin, tongue, sternum, heart, lung, and kidney.

At 48 h after injection, again, only the ^89^Zr-cetuximab-IRDye (2.0 eq) conjugate showed significant differences for blood, liver, and tumor uptake compared with ^89^Zr-cetuximab-IRDye (0 eq). Overall, blood levels (4.3 ± 3.8, 3.1 ± 1.9, 1.6 ± 0.4, and 0.8 ± 0.2%ID/g, respectively), tumor uptake (19.1 ± 6.4, 18.2 ± 7.1, 14.0 ± 3.0, and 8.1 ± 2.3%ID/g, respectively), and uptake in most normal tissues were lower than those at 24 h after injection for all conjugates. Only the liver uptake was slightly higher for each conjugate at 48 h than at 24 h (27.9 ± 3.2, 25.7 ± 8.4, 29.8 ± 1.9, and 41.8 ± 4.7%ID/g, respectively; Figure 5B). Blood, tumor, and normal tissue levels were further decreased at 72 h after injection; only the liver uptake remained about the same (Figure 5C). Tumor and liver uptake were significantly different for conjugates with 2.0 eq compared with those with 0 eq; levels of blood and of several normal organs were too low at this time point to be of any statistical value.

**Figure 6:**
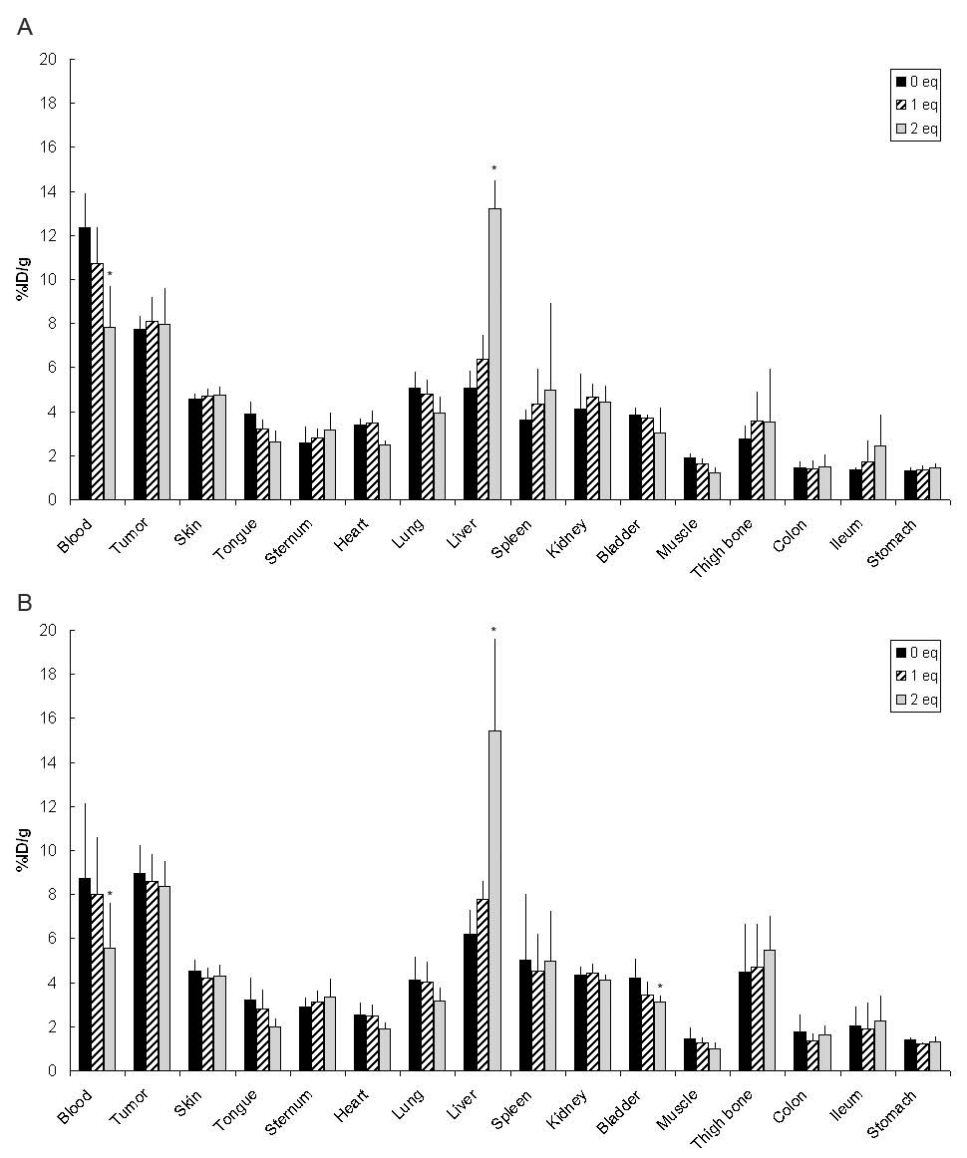
**Biodistribution of ^89^Zr-bevacizumab-IRDye800CW in tumor-bearing mice**. Biodistribution of intravenously injected ^89^Zr-bevacizumab and ^89^Zr-bevacizumab-IRDye800CW (1 and 2 eq) in FaDu xenograft-bearing nude mice at 48 (**A**) and 72 (**B**) h after injection. Bars marked with an asterisk have an uptake that is significantly (*P *≤ 0.05) different from the uptake of ^89^Zr-bevacizumab. Data are presented as %ID/g ± SD.

The biological effect of the number of dye groups coupled to a mAb was also studied for ^89^Zr-bevacizumab-IRDye800CW (0, 1.0, or 2.0 eq) in a biodistribution study in nude mice bearing FaDu tumors (Figure 6). Again, a significantly faster blood clearance was seen only for ^89^Zr-bevacizumab-IRDye800CW (2.0 eq) compared with ^89^Zr-bevacizuab-IRDye800CW (0 eq), with concomitantly significant increased liver uptake at 48 h (Figure 6A) as well as at 72 h (Figure 6B). Blood levels for conjugates with 0, 1.0, and 2.0 eq of coupled dye at 48 h were 12.4 ± 1.6, 10.7 ± 1.7, and 7.8 ± 1.9%ID/g, respectively, while liver uptake was 5.1 ± 0.8, 6.1 ± 1.1, and 13.2 ± 1.3%ID/g, respectively (Figure 6A). Tumor values were not significantly different for conjugates containing 0, 1.0, and 2.0 eq of dye: 7.8 ± 0.6, 8.1 ± 1.1, and 7.9 ± 1.7, respectively at 48 h. At 72 h (Figure 6B), blood levels were further decreased (8.7 ± 3.4, 8.0 ± 2.6, and 5.6 ± 2.1%ID/g, respectively), and liver values increased (6.2 ± 1.1, 7.8 ± 0.8, and 15.4 ± 4.2%ID/g, respectively). Tumor uptake did not show significant changes.

### Imaging

**Figure 7:**
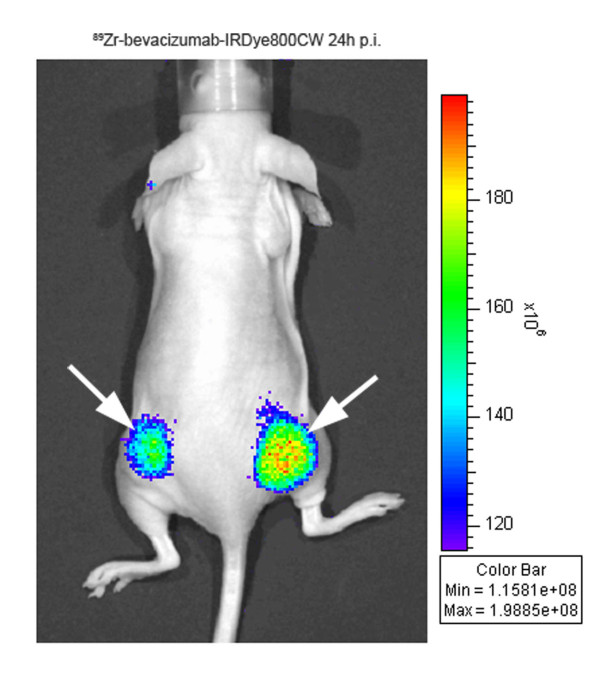
**Optical imaging with ^89^Zr-bevacizumab-IRDye800CW in a tumor-bearing mouse**. NIR image of a nude mouse bearing FaDu tumors on both lateral sides 24 h after injection of ^89^Zr-bevacizumab-IRDye800CW (1.0 eq). Tumors are indicated with white arrows.

To confirm selective tumor targeting of the ^89^Zr-mAb-IRDye800CW product with an optical imaging device, mice injected with the ^89^Zr-mAb-IRDye800CW conjugates were imaged 24, 48, and 72 h after injection before being sacrificed for biodistribution. Figure 7 shows an example of a mouse injected with ^89^Zr-bevacizumab-IRDye800CW (1.0 eq) 24 h after injection. Tumors were clearly visualized carrying 6 to 12 pmol of dye as could be estimated from the ^89^Zr tumor accumulation data at 48 h.

## Discussion

During the past years, we have developed procedures for coupling of ^89^Zr to mAbs for PET imaging. First clinical trials have indicated that ^89^Zr-immuno-PET might be an attractive tool for tumor detection and to allow better understanding of mAb therapy efficacy, more efficient mAb development, and more patient-tailored therapy [1,2]. By assuring the inert and cGMP-compliant coupling of ^89^Zr to mAbs for human use, FDA-approved mAbs like cetuximab, bevacizumab, rituximab, and trastuzumab included, ^89^Zr-immuno-PET can now be clinically applied in Europe without additional toxicology studies being required. In a comparable approach, we now aimed the inert coupling of IRDye800CW to mAbs, enabling clinical PID as a complementary tool to radioimmunodetection.

In the present study, we evaluated the impact of the coupling of different numbers of IRDye800CW molecules to cetuximab and bevacizumab on mAb integrity, immunoreactivity, and *in vivo *biodistribution. To facilitate a quantitative analysis in this study and to open possibilities for dual modal imaging in future studies, cetuximab and bevacizumab were labeled with ^89^Zr. To exclude any detrimental effect on the mAbs, just 0.5 Df group was coupled to the lysine residues of the mAbs, while our previous studies revealed that at least four Df groups can be coupled without any impairment of *in vitro *and *in vivo *mAb characteristics. Subsequent coupling of up to five IRDye800CW groups to the lysine residues of the ^89^Zr-mAbs, followed by PD10 purification, resulted in conjugates that were more than 99% pure for ^89^Zr as well as for IRDye800CW, while the integrity of the mAbs as assessed by HPLC analysis remained fully preserved. Also, the immunoreactivity remained preserved under the conditions tested. Moreover, aforementioned ^89^Zr-mAb-IRDye800CW conjugates remained stable when stored in 0.9% NaCl at 4°C and in PBS and human serum at 37°C for at least 96 h. Despite this optimal quality control [QC], an alteration in the biodistribution of ^89^Zr-cetuximab and ^89^Zr-bevacizumab was observed when more than 1 eq of IRDye800CW was coupled: blood clearance was faster, while liver uptake increased. In the case of cetuximab, tumor uptake decreased, while this phenomenon was not observed with bevacizumab. The latter might be due to the relatively high bevacizumab dose of 40 μg used in our studies, which might result in antigen saturation [35]. These data indicate that for clinical PID studies, on an average, not more than 1 eq of IRDye800CW should be coupled per mAb molecule even when no dual labeling with ^89^Zr is performed; otherwise, impairment of mAb biodistribution characteristics might occur. The mAbs with 1 eq of IRDye800CW coupled showed clear tumor delineation by optical imaging.

The use of IRDye800CW-labeled mAbs and antibody-like fragments for tumor detection has been described in several preclinical studies in mice, but to the best of our knowledge, not in clinical trials thus far [12,13,19,34,36]. In two of these studies, besides IRDye800CW, also a radioisotope was coupled to the mAb to allow a dual modality optical/nuclear (SPECT or PET) imaging [12,19]. The inertness of dye coupling was, however, not quantitatively demonstrated. Sampath et al. [12] developed and tested a trastuzumab-based conjugate containing IRDye800CW as well as indium-111, which was designated as (^111^In-diethylene triamine pentaacetic acid [DTPA])*_n_*-trastuzumab-(IRDye800CW)*_m_*. On an average, 10 DTPA chelate groups and between 7 and 10 IRDye800CW groups were coupled, far more than the critical level of 1 dye group as found in our study. Although the conjugate retained immunoreactivity *in vitro *and tumor uptake *in vivo*, a very high liver uptake was observed. In a next study of the same group, a dual-labeled conjugate suitable for PET instead of SPECT imaging was developed: (^64^Cu-1,4,7,10-tetraazacyclododecane-1,4,7,10-tetraacetic acid [DOTA])*_n_*-trastuzumab-(IRDye800)*_m _*[19]. This time, 2.4 DOTA groups and 2.2 IRDye800CW groups were coupled to the mAb. This conjugate showed good uptake in both primary and metastatic lesions as demonstrated by PET and optical imaging, but also this time, high nonspecific liver uptake was observed 24 h after injection. The authors propose the high liver uptake to originate from the interaction of the Fc portion of the antibody with hepatocytes. However, as demonstrated herein, overloading of the mAb with DOTA chelate and dye groups might well be the main culprit.

Rapid blood clearance and extensive liver accumulation have also been observed for mAbs coupled with other chemical groups to their lysine residues even under conditions that did not cause impairment of mAb immunoreactivity. Coupling of ^99m^Tc/^99^Tc-MAG3 or ^186^Re-MAG3 chelate groups to lysine residues of a mAb caused faster blood clearance when, on an average, more than 8 groups were coupled, while immunoreactivity only slightly decreased upon coupling of more than 12 groups. Concomitantly, an increased uptake of the antibody conjugates in the liver and intestines was observed [37]. For mAbs labeled with ^153^Sm via DTPA, rapid blood clearance and liver accumulation were observed in rats when 20 chelate groups were coupled per mAb [38]. A similar phenomenon was observed when photoactive dyes were coupled to the mAbs. Immunoreactivity did not alter when 19 hydrophilic fluorescein groups were coupled per mAb. However, upon evaluation of the biodistribution in mice of mAbs coupled with 4 to 14 dye groups, coupling of more than 10 dye groups per mAb resulted in enhanced blood clearance [39]. During development of conjugates for photoimmunotherapy, upon coupling of the hydrophobic photosensitizer meta-tetrahydroxyphenylchlorin [*m*THPC] to lysine residues of mAbs, a twofold higher liver uptake and almost twofold lower tumor and blood values were observed when just 0.9 *m*THPC group was coupled per mAb molecule, while four *m*THPC groups could be coupled to a mAb without a decrease in immunoreactivity [40]. These studies clearly show that depending not only on the number of chelate or dye groups, but also on their nature, alterations in hydrophobicity, charge, or conformation might be introduced, resulting in an altered behavior of the antibody conjugates *in vivo*.

Translating these findings to IRDye800CW, it seems that this relatively large molecule (molecular weight approximately 1,000 Da), having a strongly hydrophobic center and three potentially negatively charged SO_3_H-groups on the outside, apparently induces - via intramolecular tension - a conformation change of the mAb molecule. Accordingly, it appears that a mAb molecule with conformational changes induced by two dye groups is *in vivo *'recognized' by the liver as a denatured mAb and eliminated from the blood/biocirculation.

Although not the direct purpose of this study, ^89^Zr-mAb-IRDye800CW conjugates can also be used for a clinical dual modal imaging. To this end, ^89^Zr as well as the IRDye800CW should be coupled in a cGMP-compliant way to the mAb. For coupling of ^89^Zr to mAbs, we used a multistep procedure that has been developed by Verel et al. [33] using a succinylated derivative of desferrioxamine B (*N*-sucDf) as a bifunctional chelate. The choice of desferrioxamine B is attractive because it is used clinically in a safe way for many years. In addition, several clinical immuno-PET studies have been performed with ^89^Zr-labeled mAbs [1-4]. A shortcoming of the *N*-sucDf-based labeling procedure, however, is that it is relatively time-consuming and complicated; therefore, it is challenging with respect to cGMP compliancy. Recently, the newly developed *p*-isothiocyanatobenzyl derivate of desferrioxamine B (Df-Bz-NCS) was introduced, which provides an efficient and rapid preparation of ^89^Zr-mAbs. Df-Bz-NCS as well as IRDye800CW are commercially available at a cGMP quality. In complementary experiments for the production of ^89^Zr-mAb-IRDye800CW, we have shown that (1) mAb-IRDye800CW conjugates containing just 0.5 Df group per mAb can efficiently be labeled with ^89^Zr at levels of at least 74 MBq/mg, which is sufficient for clinical imaging; (2) Df-Bz-NCS is equally well efficient for ^89^Zr-labeling as *N*-sucDf, while procedures are more facile; and (3) since the *N*-sucDf route requires a pH 4.0 and 9.5 step with this chelate, ^89^Zr labeling has to be performed prior to IRDye800CW coupling, while for Df-Bz-NCS, the sequence of conjugation and labeling is not critical.

Altered expressions of EGFR and VEGF are early steps in the development of many cancers. Therefore, these are appealing targets for early photodetection of tumor cell clusters arising in the epithelial linings as well as for detection of small, established tumor nodules, which cannot be identified by current radiological and nuclear techniques. Therefore, in the present study, cetuximab (anti-EGFR) and bevacizumab (anti-VEGF) were used as the model mAbs. At our institute, PID with IRDye800CW-mAbs will be particularly explored as molecular probes for tumor detection in endoscopic procedures [41], using optical coherence tomography for obtaining structural information [42].

## Conclusion

This study describes the coupling of different equivalents of IRDye800CW to ^89^Zr-labeled cetuximab and bevacizumab and the evaluation of the conjugates in biodistribution and optical imaging studies. All conjugates showed optimal immunoreactivity and were > 95% stable in storage buffer at 4°C and 37°C and in human serum at 37°C for at least 96 h. Alteration of biodistribution was observed when more than 1 eq of IRDye800CW was coupled to the mAbs; therefore, conjugation of not more than one dye group per mAb is recommended for clinical PID studies to assure inertness of coupling.

## Competing interests

The authors declare that they have no competing interests.

## Authors' contributions

IHCdR and MAS performed the labeling and conjugation experiments and the QC analyses. MSvW performed all animal studies. RC coordinated and executed all the experiments and drafted the manuscript. GWMV reviewed the experimental data and manuscript. GAMSvD designed and coordinated the study and reviewed all the data and the manuscript. All authors read and approved the final manuscript.

## Supplementary Material

Additional file 1 **Additional methods**. Description of the radiolabeling of cetuximab/bevacizumab with ^89^Zr and the immunoreactivity assay for ^89^Zr-bevacizumab(-IRDye800CW). Click here for file
