# Supplementary material for: Inert coupling of IRDye800CW to monoclonal antibodies for clinical optical imaging of tumor targets
Source: EJNMMI Res. 2011 Dec 1;1:31. doi: 10.1186/2191-219X-1-31 (PMC3250998; doi:10.1186/2191-219X-1-31)
Supplement: Additional file 1 — Additional methods. Description of the radiolabeling of cetuximab/bevacizumab with 89Zr and the immunoreactivity assay for 89Zr-bevacizumab(-IRDye800CW). [file 2191-219X-1-31-S1.DOC]

**Additional Methods**

**Radiolabeling of cetuximab/bevacizumab with 89Zr**

The desferal chelate was succinylated (*N*-sucDf),temporarily filled with Fe(III), and coupled to the lysineresidues of cetuximab or bevacizumab by means of a tetrafluorophenol-*N*-sucDfester. An equimolar amount of ester was added to the mAb. Conjugation was performed at room temperature for 30min at pH 9.5–9.7. Thereafter, a sample was taken for HPLC analysis to determine the chelate-to-mAb ratio. The pH of the mixture wasset to pH 4.2–4.4 by use of 100 mg/mL gentisic acid, 0.25 M H2SO4, and 50 µL of 25mg/mL ethylenediaminetetraacetic acid (EDTA; Calbiochem)were added to remove Fe(III) from the Df-chelate. The solution was incubatedfor 30 min at 35°C. Nonconjugated chelate, Fe(III) and EDTA were removed by size exclusion chromatography using a PD10 column and 0.9% NaCl as eluent. The flow through and the first 1.5 mL were discarded. The next 2 mL contained the modified antibody. Subsequently, labeling was performed with 50-100 MBq of 89Zr. To this end, 2 M Na2CO3 was added to the 89Zr-oxalate solution to adjust the pH, and mixed for 3 min. The pH of the solution was set to pH 6.7-6.9 with 0.25 M 4-(2-hydroxyethyl)-1-piperazineethanesulfonicacid (HEPES; Sigma-Aldrich, St. Louis, MO) buffer. *N*-sucDf-cetuximab/bevacizumab was added andthe mixture was incubated for 60 min at room temperature. The total reaction volume of 2 ml 89Zr-*N*-sucDf-cetuximab/bevacizumab was purified on a PD10 column using 0.9% NaCl as eluent. The flow through and the first 1.5 mL were discarded. The next 2 mL containing the 89Zr-labeled mAb were collected and a sample was used for HPLC analysis to determine the mAb concentration.

**Immunoreactivity assay for 89Zr-bevacizumab(-IRDye800CW)**

MaxiSorpTM break apart 96-wells plates (Nunc GmbH & Co. KG, Langenselbond, Deutschland) were coated with recombinant human VEGF165 (R&D Systems Europe Ltd, Abingdon, United Kingdom), dissolved in 1% BSA/PBS. VEGF was diluted to a concentration of 1 µg/mL with coating buffer, a mixture of Na2CO3, NaHCO3 and NaN3­ in sterile water for injection, pH 9.3-9.8. 50 µL of the solution were added to the wells (50 ng/well) and incubated for at least 20 h at 4°C. The wells were washed with subsequently 0.05% tween20 in PBS (3 times 150 μL) and PBS (2 times 150 μL), and blocked with 150 μL 4% BSA/PBS at RT for 2 h to prevent non-specific binding. 89Zr-bevacizumab and 89Zr-bevacizumab-IRDye800CW (1 and 2 equivalents) were diluted in PBS (concentrations, 350 - 2.7 ng/mL), added to the wells, and incubated by shaking at RT overnight. Competition experiments were performed by diluting bevacizumab standard solution (25 mg/mL) with PBS and adding an excess of unlabeled bevacizumab up to 500-fold. Subsequently, the reaction solution and 3 wash steps of 150 µL PBS were collected from the wells. Both the radioactivity in the antigen-coated wells and the collected reaction and wash solution containing unbound radiolabeled mAb were counted with an LKB-1282-Compugamma-system.
